# Chlorophyll fluorescence characteristics and H_2_O_2_ contents of Chinese tallow tree are dependent on population origin, nutrients and salinity

**DOI:** 10.1093/aobpla/plae024

**Published:** 2024-05-02

**Authors:** Mengyue He, Lihong Ge, Xue Hui, Wenrao Li, Jianqing Ding, Evan Siemann

**Affiliations:** School of Life Sciences, Henan University, Kaifeng, Henan Province 475004, China; School of Life Sciences, Henan University, Kaifeng, Henan Province 475004, China; School of Life Sciences, Henan University, Kaifeng, Henan Province 475004, China; School of Life Sciences, Henan University, Kaifeng, Henan Province 475004, China; School of Life Sciences, Henan University, Kaifeng, Henan Province 475004, China; Department of Biosciences, Rice University, Houston, TX 77005, USA

**Keywords:** Antioxidant defences, Chinese tallow tree, N:P ratio, nutrient deficiency, PSI and PSII chlorophyll fluorescence characteristics, salinity

## Abstract

Plants from invasive populations often have higher growth rates than conspecifics from native populations due to better environmental adaptability. However, the roles of improved chlorophyll fluorescence or antioxidant defenses in helping them to grow better under adverse situations are insufficient, even though this is a key physiological question for elucidating mechanisms of plant invasion. Here, we conducted experiments with eight native (China) and eight introduced (USA) populations of Chinese tallow tree (*Triadica sebifera*). We tested how salinity, nutrients (overall amount or N:P in two separate experiments) and their interaction affected *T. sebifera* aboveground biomass, leaf area, chlorophyll fluorescence and antioxidant defenses. Plants from introduced populations were larger than those from native populations, but salinity and nutrient shortage (low nutrients or high N:P) reduced this advantage, possibly reflecting differences in chlorophyll fluorescence based on their higher PSII maximum photochemical efficiency (*F*_v_/*F*_m_) and PSI maximum photo-oxidizable P700 in higher nutrient conditions. Native population plants had lower *F*_v_/*F*_m_ with saline. Except in high nutrients/N:P with salinity, introduced population plants had lower electron transfer rate and photochemical quantum yield. There were no differences in antioxidant defenses between introduced and native populations except accumulation of hydrogen peroxide (H_2_O_2_), which was lower for introduced populations. Low nutrients and higher N:P or salinity increased total antioxidant capacity and H_2_O_2_. Our results indicate that nutrients and salinity induce differences in H_2_O_2_ contents and chlorophyll fluorescence characteristics between introduced and native populations of an invasive plant, illuminating adaptive mechanisms using photosynthetic physiological descriptors in order to predict invasions.

## Introduction

Successful invasive plants often optimize their performance by evolving broad environmental tolerance, physiological characteristics or high phenotypic plasticity, including larger leaf area or specific leaf area and greater net photosynthesis rate compared with non-invasive native or alien species when faced with limited nutrient availability (Funk and Vitousek 2007; Funk 2013; Ordonez and Olff 2013; Pintó-Marijuan and Munné-Bosch 2013; Yang *et al.* 2015a; Morris *et al.* 2020; Li *et al.* 2023). However, the mechanisms that promote these dominant physiological characteristics under various abiotic conditions remain poorly understood (Funk and Vitousek 2007; Funk 2013; Pintó-Marijuan and Munné-Bosch 2013; Li *et al.* 2023).

Chlorophyll fluorescence characteristics can highlight the influence of environmental resources on photosynthesis and plant growth (Misra *et al.* 2012; Huang *et al.* 2015; Jiao *et al.* 2017; Deng *et al.* 2019). For instance, when faced with water stress or nutrient shortage, invasive species can exhibit changes in PSII maximum photochemical efficiency (*F*_v_/*F*_m_). For example, *Juniperus virginiana* maintained its initial *F*_v_/*F*_m_ level under water stress (Bihmidine *et al.* 2010), while *Melinis minutiflora* experienced a decrease in *F*_v_/*F*_m_ (Musso *et al.* 2021). Interestingly, fertilizer application did not affect the non-photochemical quenching coefficient (qN) (Musso *et al.* 2021). In the case of *Triadica sebifera*, introduced populations showed a higher PSII effective quantum yield (Y(II)) but lower *F*_v_/*F*_m_ compared with native populations when grown in sterilized soil (Deng *et al.* 2019). Conversely, under high water stress conditions, invasive *M. minutiflora* exhibited higher *F*_v_/*F*_m_ than native *Schizachyrium microstachyum*, and this difference was reduced with fertilization (Musso *et al.* 2021). Furthermore, the growth response and chlorophyll fluorescence characteristics of species can be influenced by different N:P ratios in the environment (Güsewell 2004; Li and Sun 2016). In a study by Jiao *et al.* (2017), reversible inactivation of PSII was observed in invasive *Chlorella vulgaris* under non-phosphorus control, as measured by chlorophyll fluorescence characteristics. These findings indicate that the performance of chlorophyll fluorescence in both introduced and native plants can reflect their ability to adapt to variations in resource availability, and may serve as a predictive tool in the case of new invasions.

Photosystem II is more sensitive to environmental conditions such as nutrient deficiency than PSI, which is considered relatively stable (Rochaix 2011; Tikkanen *et al.* 2014; Huang *et al.* 2015). Similar to PSII variables, PSI electron transfer (ETR(I)), PSI effective quantum yield (Y(I)) and PSI maximum photo-oxidizable P700 (*P*_m_) suggest the improvement or inhibition of PSI electron transport may be due to environmental variables (Shimakawa and Miyake 2018). However, studies on introduced and native populations of invasive plants, particularly in terms of the simultaneous determination of PSI and PSII in response to different nutrient availabilities, are lacking.

Another important factor to consider with regard to electron transport in plants experiencing abiotic stresses, such as nutrient deficiency, is the excessive formation of reactive oxygen species (ROS) (Sharma *et al.* 2012; Bose *et al.* 2014; Mittler *et al.* 2022). Nutrient deficiency can disrupt plant growth and development by disturbing homeostasis and ion distribution within plant cells, causing ROS imbalance and affecting ROS production, accumulation, scavenging and transport (Mittler *et al.* 2022). Plants have developed several protective mechanisms to safeguard chloroplasts (Takahashi and Badger 2011; Huang *et al.* 2015). One such mechanism is enhancing qN, which is a physical process used to dissipate heat generated by excessive light energy and reduce ROS accumulation. Another mechanism is strengthening antioxidant substance activities or expression to scavenge ROS through chemical reactions. For instance, the superoxide anion radical ($O_{2}^{-}\cdot$) is converted to hydrogen peroxide (H_2_O_2_) through the action of superoxide dismutase (SOD). This process can potentially lead to the formation of the hydroxyl radical (•OH) (Sharma *et al.* 2012; Mittler *et al.* 2022). SOD activity differs between native (*S. microstachyum*) and invasive (*M. minutiflora*) grasses under nutrient deficiency scenarios, suggesting that their oxidative responses may differ (Musso *et al.* 2021). Notably, when fertilized, SOD activity is higher for *S. microstachyum* than for the invasive *M. minutiflora*. Therefore, the ROS accumulation and scavenging ability accurately reflect the resistance and self-protection abilities against abiotic stress (Pintó-Marijuan and Munné-Bosch 2013; Mittler *et al.* 2022).

A number of studies have identified salt-induced reductions in photosynthesis as a result of negative effects on photosynthetic electron transport and the positive induction and production of ROS in plants (Pintó-Marijuan and Munné-Bosch 2013; Zhang *et al.* 2016; Pan *et al.* 2021). However, the effect of salinity on PS function is dependent on salt concentration, stress exposure time and plant species (Zahra *et al.* 2022). There have also been reports suggesting that mild salinity can act as an external stimulus, aiding plants in tolerating other stresses and promoting growth (Liu *et al.* 2023). Salinity may not only alter the *F*_v_/*F*_m_ of plants but also help them resist low temperature stress by increasing the contents of chlorophyll and carotenoids (Liu *et al.* 2023), as well as enhancing low-temperature-induced antioxidant capacity (Li *et al.* 2015). Increased SOD activity in response to salinity has been documented in numerous invasive plants (Pintó-Marijuan and Munné-Bosch 2013; Zhang *et al.* 2016). Additionally, competition between salt ions and various nutrients can easily lead to mineral nutrient stress. However, further research is needed to clarify the roles of salt under different nutrient availability conditions in introduced versus native populations of invasive plants.

Chinese Tallow tree (*T. sebifera*), native to China, was first introduced to Georgia ~250 years ago and is now invasive throughout the southeastern USA (Pile *et al.* 2017). Common garden experiments showed that introduced populations of *T. sebifera* grow faster, with significantly larger shoot biomass and leaf area compared with plants from native populations (Siemann and Rogers 2003; Zou *et al.* 2007; Yang *et al.* 2015a, 2022; Li *et al.* 2020). Additionally, compared to native *T. sebifera* populations, invasive populations showed lower photosynthetic carbon cost and higher photosynthetic rates (Zou *et al.* 2007; Li *et al.* 2020). Studies also showed that the growth advantages of plants from introduced populations persist under saline conditions (Yang *et al.* 2015b, 2022). However, the physiological adaptability of introduced versus native populations to salinity and diverse nutritional environments remains unexplored. This knowledge gap hinders our comprehensive understanding of the invasion mechanisms.

Here, we measured chlorophyll fluorescence characteristics and antioxidant parameters in introduced and native populations of *T. sebifera*. Our aim was to determine if these traits vary among introduced and native populations under different nutrient environmental conditions. Specifically, we addressed the following questions: (i) How do the chlorophyll fluorescence characteristics and antioxidant capacity of *T. sebifera* respond to nutrient deficiency or high N:P ratio? (ii) How does salinity change these responses? (iii) Are these traits associated with the adaptations of introduced populations? The research findings will reveal the superior physiological traits that invasive plants have evolved, and by utilizing these characteristics, we can make preliminary assessments of a plant’s invasive availability.

## Materials and Methods

### Material selection and cultivation

In the fall of 2018, we collected seeds from healthy adult trees in eight populations within the native range and eight populations within the introduced range [**see Supporting Information—**Table S1]. We randomly combined mature seeds from several trees (6–10, widely spaced) within each population. We removed the waxy coatings on the seeds by soaking them in soapy water and scrubbing them. Then we surface sterilized them with dilute bleach and placed them in moist sand at 4 °C for 2–3 months. For a more detailed description, please refer to Li *et al.* (2020).

In May 2019, we sowed ~200 seeds from each population in a tray filled with a mixture of vermiculite and commercial topsoil that we placed in an unheated, open-sided greenhouse located in Kaifeng, China. We kept seeds and seedlings well watered.

Once seedlings had four leaves, we transferred them individually to PVC tubes (height = 24.5 cm, diameter = 6.3 cm) filled with 1000 g of dry, low-nutrient soil (total carbon: 6.43 g kg^−1^; total nitrogen: 0.002 g kg^−1^; available phosphorus: 0.82 mg kg^−1^). We placed the PVC tubes within the experimental area and repositioned them every 2 weeks during the growth period to ensure they received sufficient and uniform light.

### Experimental designs

The nutrient deficiency and salinity experiment investigated the effects of nutrient levels (normal vs. deficient) and salinity (saline or not) on introduced vs. native tallow populations (eight of each) in a factorial experiment. We watered normal nutrient seedlings with 1/2 Hoagland’s nutrient solution (*N* = 7.5 mM, *P* = 1 mM, *K* = 3.5 mM plus micronutrients) and the nutrient-deficient seedlings with water. We added 5 g L^−1^ of NaCl to seedlings in saline treatments. In total, there were 448 seedlings (8 introduced populations and 8 native populations × 2 nutrient levels × 2 salinity levels × 7 replicates).

The N:P and salinity experiment investigated the effects of N:P ratios (15:2 vs. 15:0.2) and salinity (saline or not) on introduced vs. native tallow populations (8 of each) in a factorial experiment. We watered 15:2 nutrient (*N* = 15 mM, *P* = 2 mM, *K* = 7 mM plus micronutrients) and 15:0.2 nutrient seedlings (*N* = 15 mM, *P* = 0.2 mM, *K* = 7 mM plus micronutrients) with modified Hoagland’s nutrient solutions. We added 5 g L^−1^ of NaCl to seedlings in saline treatments. In total, there were 448 seedlings (8 introduced populations and 8 native populations × 2 N:P levels × 2 salinity levels × 7 replicates).

In each of the experiments, we used four replicates to measure biomass and leaf area. We used three of these four replicates to also measure chlorophyll fluorescence characteristics. We used the remaining three replicates to measure antioxidant defenses.

### PS II and PS I parameter measurements

Three days prior to harvest, from 8:00 to 15:00, we determined chlorophyll fluorescence parameters using a portable modulated chlorophyll fluorometer (DUAL-PAM-100, Heinz Walz GmbH, Effeltrich, Germany) on the top 3 or 4 fully expanded leaves. Before measurement, we performed a dark adaptation treatment on the leaves for 30 min. We measured chlorophyll fluorescence parameters using the ‘SP-Analysis’ and ‘P700+Fluor’ Dual Channel modes, following the methods outlined by Huang *et al.* (2015).

To estimate the activity of photosystem II (PSII), we turned the measuring light on (less than 1 μmol m^−2^ s^−1^) and recorded the minimum fluorescence (*F*_0_) after dark adaptation. This was followed by a saturation pulse (300 ms and 10 000 μmolm^−2^ s^−1^), which interrupted the PSII reaction, and we recorded the maximum fluorescence (*F*_m_) after dark adaptation. We calculated PSII maximum photochemical efficiency (*F*_v_/*F*_m_) using the formula *F*_v_/*F*_m_  = (*F*_m_−*F*_0_)/*F*_m_, as described by Kitajima and Butler, (1975).

Next, we turned on the actinic light irradiation and gave a saturation pulse (300 ms and 10 000 μmol m^−2^ s^−1^) to record the chlorophyll fluorescence peak (${F^{\prime }}_{m}$) and steady-state fluorescence after light adaptation (*F*). Simultaneously, fluorescence quenching occurs and we obtained the photochemical quenching coefficient (qP = $\left({F^{\prime }}_{m}-F)/({F^{\prime }}_{m}-{F^{\prime }}_{0}\right)$) and non-photochemical quenching coefficient (qN = $1-({F^{\prime }}_{m}-{F^{\prime }}_{0})/(F_{m}-F_{0})$) according to van Kooten and Snel (1990). We calculated the PSII photochemical quantum yield (Y(II)) and PSII electron transfer rate (ETR(II)) according to Y(II) = $({F^{\prime }}_{m}-F)/{F^{\prime }}_{m}$ (Harbinson *et al.* 1989) and ETR(II) = PAR (photosynthetic active radiation, 962 μmol m^−2^ s^−1^) × 0.85 × 0.5 × Y(II), respectively. Here, 0.5 represents the proportion of absorbed light reaching photosystem I (PSI) or PSII, and 0.85 is the absorbed irradiance considered as 0.85 of the incident irradiance (Huang *et al.* 2015).

We first recorded the PSI maximum fluorescence signal (*P*_m_) after dark adaptation to estimate the amount of photo-oxidizable PSI reaction centres. We then determined ${P^{\prime }}_{m}$ using a saturation pulse (300 ms and 10 000 μmol m^−2^ s^−1^) and actinic light irradiation (962 μmol m^−2^ s^−1^), similar to the process used for *P*_*m*_. We recorded or calculated the parameters for PSI photochemical quantum yield (Y(I)), and PSI electron transfer rate (ETR(I)) in a similar way as Y(II) or ETR(II), as described by Tikkanen *et al.* (2014) and Huang *et al.* (2015).

### Mass and leaf area

We removed the leaves of the seedlings and weighed them immediately. We determined the leaf area using a portable leaf area instrument (CI-203, CID, USA). We cut stems at ground level and weighed them to obtain the stem biomass.

### Antioxidant parameters

We removed the top fully expanded 3–6 leaves from the set of plants, immediately put them into liquid nitrogen, took them back to the laboratory and stored them in a −80 °C refrigerator for determination of enzyme activity and ROS contents, scavenging rate and other antioxidant parameters. We determined the total antioxidant capacity (T-AOC), SOD activity, H_2_O_2_ contents, $O_{2}^{-}\cdot$ contents and scavenging rate of hydroxyl radical (${\text{S}}_{\cdot OH}$) using Comin Biochemical Test Kits (Comin Biotechnology Co., Ltd., Suzhou, China) following the manufacturer’s instructions.

### Statistical analyses

We conducted ANOVAs (PROC Mixed, SAS 9.4) to analyse the effects of treatments, as well as the interactions between treatments, on various parameters including biomass, leaf area, chlorophyll fluorescence characteristics and antioxidant parameters. The fixed factors considered in this analysis were treatments for nutrient deficiency/low phosphorus, salinity stress and population origin. The population nested in origin was considered as a random factor. To elucidate the relationship between antioxidant parameters and chlorophyll fluorescence parameters of PSI and PSII, we employed Pearson correlation and linear regression fitting. For all measurement values, we performed Pearson correlation, while for mean values, linear regression fitting was used. This was done to differentiate the adaptive strategies between native and introduced populations under distinct treatment conditions.

## Results

### Nutrient deficiency and salinity experiment

#### Mass parameters.

Leaf, stem and aboveground mass depended on the interaction of origin, nutrients and salinity with leaf mass for US populations higher in high nutrients but lower compared to China populations in low nutrients along with a negative effect of salinity (Table 1, Supporting Information—Fig. S1A). US populations always had higher stem mass, aboveground mass and leaf area with the greatest differences in high nutrients, especially in well-watered treatments when plants were the largest (Supporting Information—Fig. S1B–D).

**Table 1.** The statistical results of nutrient deficit and water treatment experiment (experiment 1).

Variable	DF	Nutrients		Salinity		Nutrients × salinity		Origin		Origin × nutrients		Origin × salinity		O × N × S		Pop (origin)	
		*F*_1,DF_	*P*	*F*_1,DF_	*P*	*F*_1,DF_	*P*	*F*_1,14_	P	*F*_1,DF_	*P*	*F*_1,DF_	*P*	*F*_1,DF_	*P*	*Z*	*P*
**Mass variables**																	
Leaf mass (g)	231	**1069.03**	**<0.0001**	**76.49**	**<0.0001**	**50.00**	**<0.0001**	**80.96**	**<0.0001**	**258.38**	**<0.0001**	**22.88**	**<0.0001**	**22.60**	**<0.0001**	**2.10**	**0.0178**
Stem mass (g)	231	**859.43**	**<0.0001**	**70.76**	**<0.0001**	**38.65**	**<0.0001**	**111.58**	**<0.0001**	**317.35**	**<0.0001**	**28.25**	**<0.0001**	**20.58**	**<0.0001**	**2.26**	**0.0118**
Aboveground mass (g)	231	**1255.93**	**<0.0001**	**93.59**	**<0.0001**	**58.01**	**<0.0001**	**101.85**	**<0.0001**	**346.09**	**<0.0001**	**30.67**	**<0.0001**	**27.52**	**<0.0001**	**2.21**	**0.0134**
Leaf area (cm^2^)	229	**660.59**	**<0.0001**	**204.89**	**<0.0001**	**222.94**	**<0.0001**	**134.74**	**<0.0001**	**279.41**	**<0.0001**	**91.88**	**<0.0001**	**134.06**	**<0.0001**	**2.36**	**0.0092**
**PSI variables**																	
*P*_m_	168	**16.11**	**<0.0001**	**22.74**	**<0.0001**	1.55	0.2149	0.14	0.7164	**31.65**	**<0.0001**	0.32	0.5724	2.44	0.1203	**1.88**	**0.0301**
Y(I)	168	**636.07**	**<0.0001**	1.57	0.2115	0.02	0.8774	**5.58**	**0.0331**	**18.55**	**<0.0001**	**11.18**	**0.0010**	**19.07**	**<0.0001**	1.52	0.0640
ETR(I)	168	**636.57**	**<0.0001**	1.55	0.2145	0.03	0.8711	**5.56**	**0.0334**	**18.56**	**<0.0001**	**11.21**	**0.0010**	**19.05**	**<0.0001**	1.53	0.0635
**PSII variables**																	
*F*_v_/*F*_m_	168	**36.65**	**<0.0001**	1.59	0.2093	**11.1**	**0.0011**	0.59	0.4565	**15.8**	**0.0001**	0.45	0.5010	0.79	0.3748	2.00	0.0229
Y(II)	168	**472.17**	**<0.0001**	1.26	0.2632	2.16	0.1435	**6.32**	**0.0248**	**13.34**	**0.0003**	**12.4**	**0.0006**	**16.66**	**<0.0001**	1.35	0.0884
ETR(II)	168	**473.12**	**<0.0001**	1.31	0.2546	2.19	0.1406	**6.29**	**0.0251**	**13.29**	**0.0004**	**12.34**	**0.0006**	**16.61**	**<0.0001**	1.36	0.0874
qN	168	0.15	0.7007	0.21	0.6469	**18.84**	**<0.0001**	**10.94**	**0.0052**	0.86	0.3537	0.06	0.8145	0.21	0.6467	1.48	0.0699
qP	168	**470.72**	**<0.0001**	0.22	0.6371	0.03	0.8591	4.30	0.0571	**8.38**	**0.0043**	**11.55**	**0.0008**	**14.63**	**0.0002**	**1.78**	**0.0377**
**Antioxidant variables**																	
T_AOC (U g^−1^)	170	**124.92**	**<0.0001**	**13.62**	**0.0003**	0.04	0.8411	1.37	0.2621	1.34	0.2493	0.82	0.3674	1.07	0.3030	**2.05**	**0.0201**
SOD (U g^−1^)	170	**21.76**	**<0.0001**	0.12	0.7340	0.02	0.8903	1.74	0.2087	0.31	0.5804	0.02	0.8903	0.01	0.9093	**1.67**	**0.0477**
H_2_O_2_ (µmol g^−1^)	170	**95.92**	**<0.0001**	**17.08**	**<0.0001**	1.36	0.2453	**6.89**	**0.0200**	2.65	0.1051	0.02	0.6531	0.91	0.3426	1.32	0.0928
$O_{2}^{-}\cdot$ (nmol g^−1^)	170	**64.78**	**<0.0001**	**47.08**	**<0.0001**	**17.44**	**<0.0001**	2.35	0.1477	0.34	0.5592	0.94	0.3325	0.07	0.7966	**1.70**	**0.0445**
$S_{.OH}$ (%)	170	**8.97**	**0.0032**	0.010	0.9243	2.74	0.0997	**4.63**	**0.0493**	0.71	0.4007	1.44	0.2321	3.63	0.0585	**1.85**	**0.0324**

Significant results are shown in bold.

#### PSI parameters.

**Figure 1.**
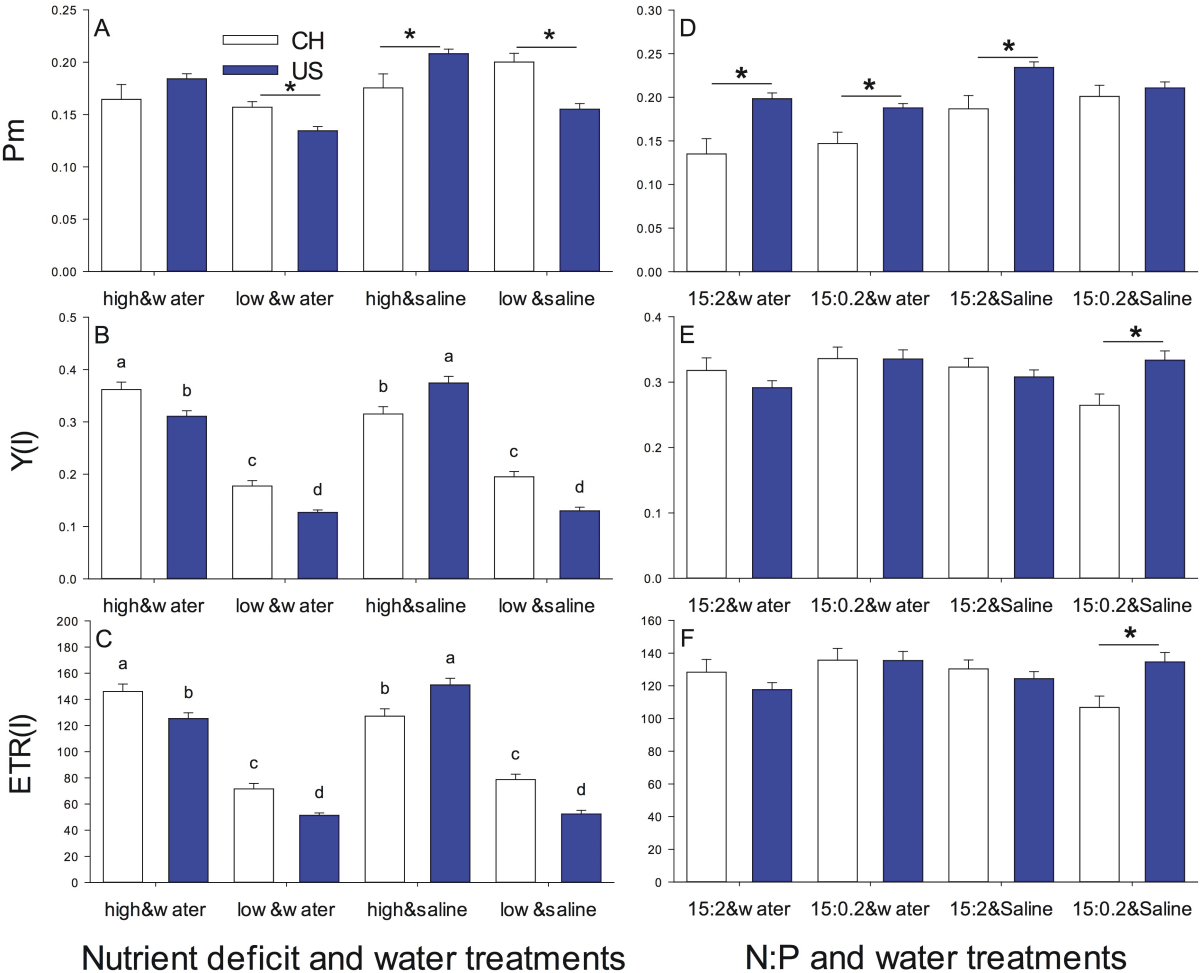
The *P*_m_ (A and D), Y(I) (B and E) and ETR(I) (C and F) in introduced (US, dark) and native (CH, light) populations in nutrient deficiency and water treatment experiment (experiment 1) (A–C) and N:P and water treatment experiment (experiment 2) (D–F). Asterisk represents significant differences between USA (introduced) and CH (native) populations at the same treatment level. Fig. A, D, E and F unmarked the letters because the interactions between treatments were not significant.

**Figure 2.**
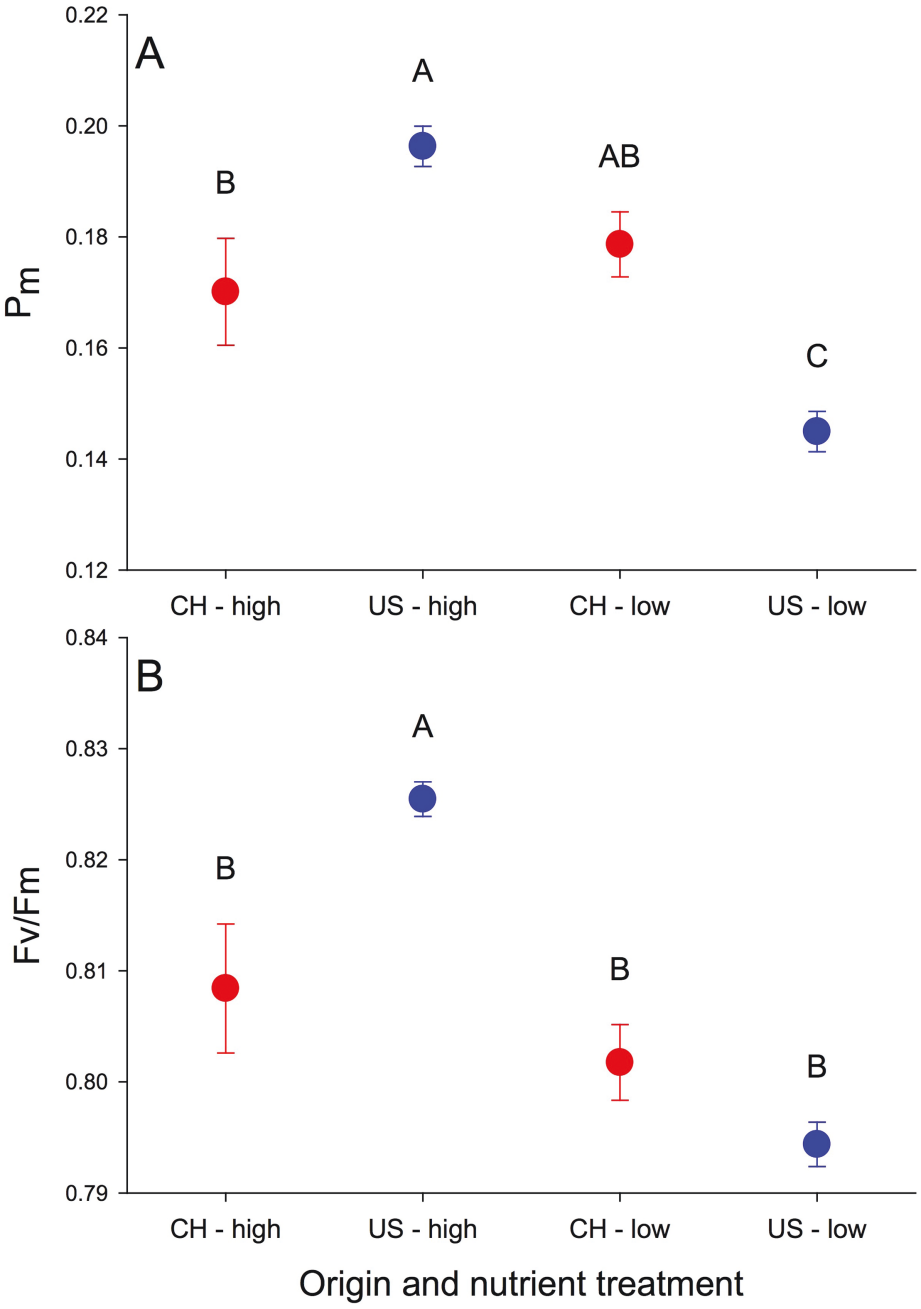
Effect of origin and nutrient treatment on *P*_m_ (A) and *F*_v_ /*F*_m_ (B) in introduced and native populations in nutrient deficit and water treatment experiment (experiment 1).

US populations had higher *P*_m_ at high nutrients but lower *P*_m_ at low nutrients than China populations because nutrient deficiency only strongly decreased *P*_m_ of US populations (Table 1, origin × nutrients; Fig. 1A and Fig. 2A). The rest of the PSI parameters all depended on the interaction of origin, nutrients and salinity (Table 1). For Y(I) and ETR(I), this reflected decreases with low nutrients coupled with a switch from China populations greater than US ones in well water or salinity and low nutrients to US populations higher than China populations in salinity and high nutrients (Fig. 1B and C).

#### PSII parameters.

**Figure 3.**
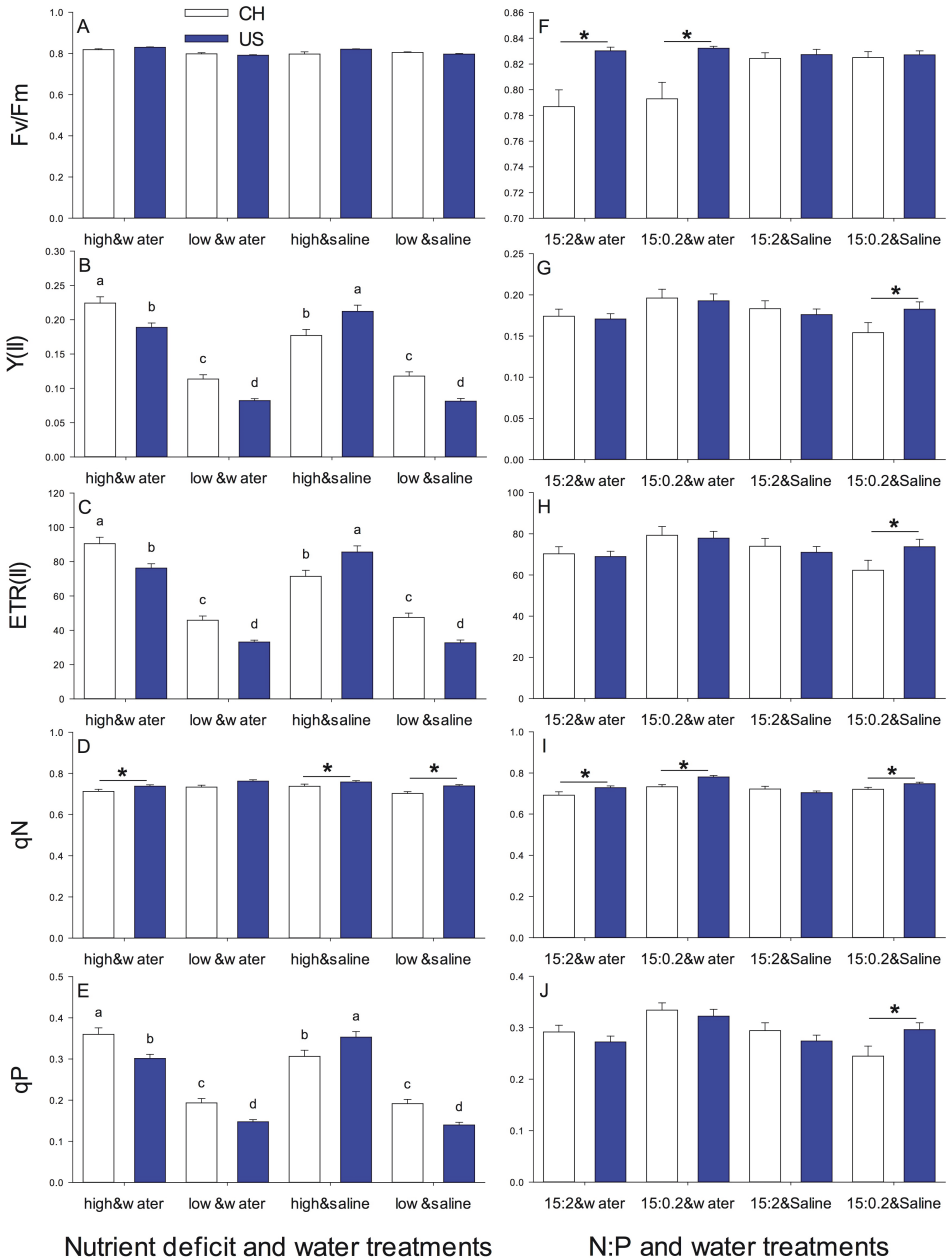
The *F*_v_/*F*_m_ (A and F), Y(II) (B and G), ETR(II) (C and H), qN (D and I) and qP (E and J) in introduced (US, dark) and native (CH, light) populations in nutrient deficit and water treatment experiment (experiment 1) (A–E) and N:P and water treatment experiment (experiment 2) (F–J). Asterisk represents significant differences between USA (introduced) and CH (native) populations at the same treatment level. Fig. 3A, D, F, G, H, I and J unmarked the letters because the interactions between treatments were not significant.

*F*_v_/*F*_m_ was higher for US populations in high nutrients compared to other origins × nutrient treatment combinations (Fig. 2A) and it was highest with high nutrients in water, lowest with low nutrients in water and intermediate with salinity (Table 1, Fig. 3A). Y(II) and ETR(II) each depended on origin × nutrients × salinity with the same patterns for means as these parameters in PSI (Table 1; Fig. 3B and C) and qP also had this pattern (Fig. 3E). qN was higher for USA vs. native populations (0.75 ± 0.01 vs. 0.72 ± 0.01) and it was lower with low nutrients in saline or high nutrients with water compared to the other nutrient × salinity treatment combinations (Table 1, Fig. 3D).

#### AOX parameters.

**Figure 4.**
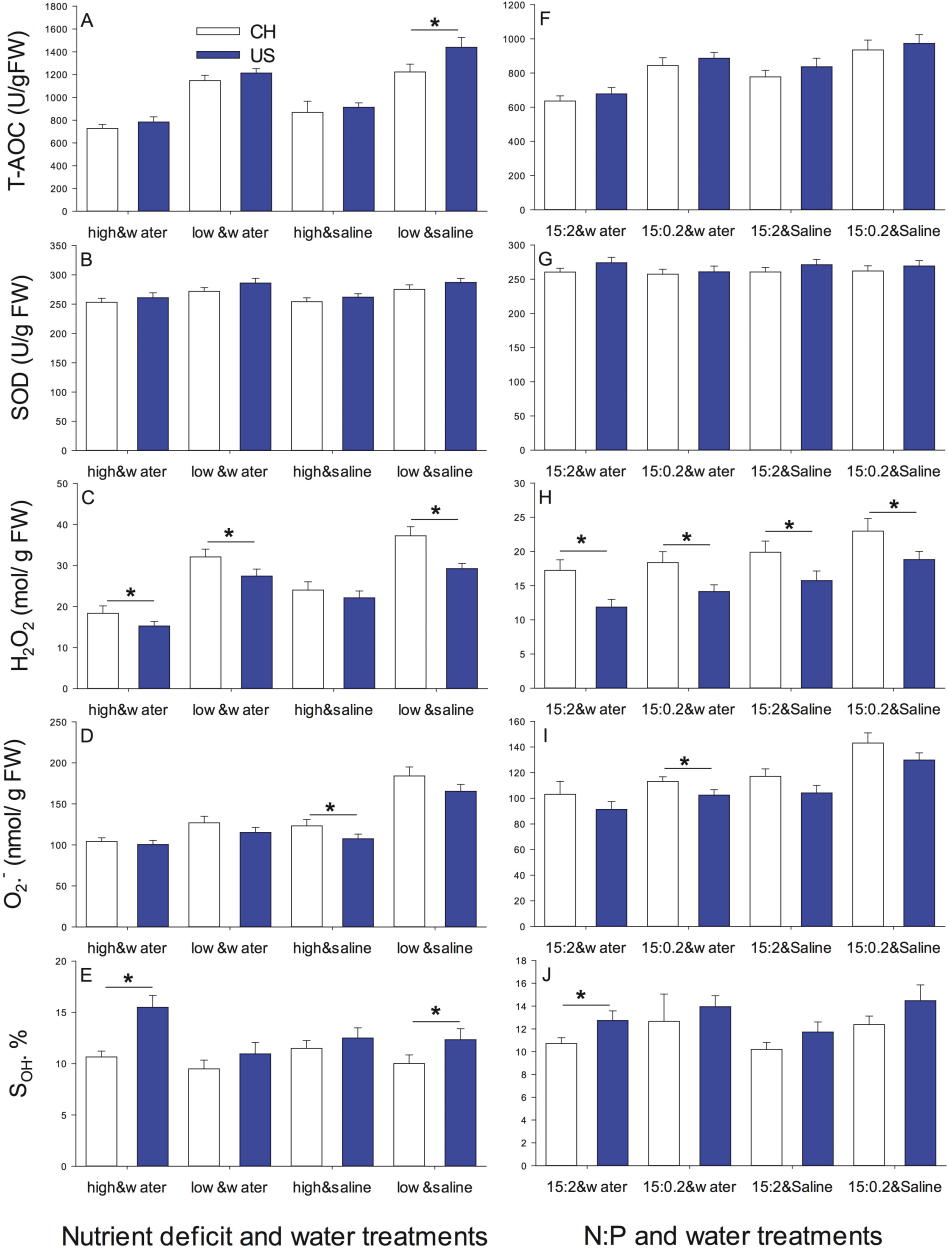
The T-AOC (A and F), SOD (B and G), H_2_O_2_ (C and H), $O_{2}^{-}\cdot$ (D and I) and ${\text{S}}_{\cdot OH}$ (E and J) in introduced (US, dark) and native (CH, light) populations in nutrient deficit and water treatment experiment (experiment 1) (A–E) and N:P and water treatment experiment (experiment 2) (F–J). Asterisk represents significant differences between USA (introduced) and CH (native) populations at the same treatment level. Fig. 4A–J unmarked the letters because the interactions between treatments were not significant.

T-AOC increased with low nutrients or salinity (Table 1, Fig. 4A). SOD increased with low nutrients (Table 1, Fig. 4B). H_2_O_2_ was higher for China populations (27.95 ± 1.22 vs. 23.54 ± 0.90 mol g^−1^ FW), at low nutrients or salinity (Table 1, Fig. 4C). $O_{2}^{-}\cdot$ increased with salinity, especially with low nutrients (Table 1, nutrients × salinity; Fig. 4D). ${\text{S}}_{\cdot OH}$ was higher with high nutrients and for US populations (China: 10.41 ± 0.38 %, USA: 12.82 ± 0.56 %) (Table 1, Fig. 4E).

#### Correlations among variables.

All the mass variables were strongly positively correlated with each other. Mass variables were most strongly positively correlated with PSI variables Y(I) and ETR(I), PSII variables *F*_v_/*F*_m_, Y(II), ETR(II) and qP. Mass variables were most strongly negatively correlated with the antioxidant variables T-AOC, H_2_O_2_ and $O_{2}^{-}\cdot$ Y(I) and ETR(I) were all strongly positively correlated. *F*_v_/*F*_m_, Y(II) and ETR(II) were strongly positively correlated. The antioxidant variables, T-AOC, SOD, H_2_O_2_ and $O_{2}^{-}\cdot$ were generally negatively correlated with PSI and PSII variables.

### N:P and salinity experiment

#### Mass parameters.

Leaf, stem and aboveground mass plus leaf area all depended on the interaction of origin, nutrients and salinity with leaf mass for US populations always higher (Table 2, Supporting Information—Fig. S1E–H). All of these variables were lower at NP 15:0.2 or salinity for the USA but they were always low for China populations so the differences between US and China populations were highest at NP 15:2 in water and lowest at NP 15:0.2 with salinity (Supporting Information—Fig. S1E–H).

**Table 2.** The statistical results of N:P and water treatment experiment (experiment 2).

Variable	DF	NP		Salinity		NP × salinity		Origin		Origin × NP		Origin × salinity		O × N × S		Pop (origin)	
		*F*_1,DF_	*P*	*F*_1,DF_	*P*	*F*_1,DF_	*P*	*F*_1,14_	*P*	*F*_1,DF_	*P*	*F*_1,DF_	*P*	*F*_1,DF_	*P*	*Z*	*P*
**Mass variables**																	
Leaf mass (g)	233	2.08	0.1504	**122.58**	**<0.0001**	1.85	0.1749	**139.33**	**<0.0001**	**20.07**	**<0.0001**	**35.79**	**<0.0001**	0.11	0.7361	**2.23**	**0.0128**
Stem mass (g)	232	0.84	0.3606	**164.32**	**<0.0001**	0.75	0.3864	**212.31**	**<0.0001**	**5.45**	**0.0204**	**80.9**	**<0.0001**	0.03	0.8641	**2.22**	**0.0132**
Aboveground mass (g)	233	0.62	0.4330	**166.07**	**<0.0001**	1.71	0.1929	**173.38**	**<0.0001**	**18.53**	**<0.0001**	**57.42**	**<0.0001**	0.06	0.8040	**2.28**	**0.0114**
Leaf area (cm^2^)	231	**10.44**	**0.0014**	**117.38**	**<0.0001**	1.98	0.1608	**278.29**	**<0.0001**	**26.75**	**<0.0001**	**64.24**	**<0.0001**	0.06	0.8008	1.62	0.0521
**PSI variables**																	
Pm	168	0.08	0.7821	**32.36**	**<0.0001**	0.15	0.6975	**6.65**	**0.0219**	**4.53**	**0.0347**	2.33	0.1284	0.28	0.5963	**2.06**	**0.0197**
Y(I)	168	0.52	0.4718	1.51	0.2203	**5.44**	**0.0208**	0.29	0.5985	**7.25**	**0.0078**	3.78	0.0537	2.02	0.1575	1.03	0.1516
ETR(I)	168	0.52	0.4705	1.53	0.2183	**5.45**	**0.0208**	0.29	0.5995	**7.24**	**0.0078**	3.79	0.0531	2.02	0.1574	1.04	0.1500
**PSII variables**																	
*F*_v_/*F*_m_	168	0.23	0.6298	**11.44**	**0.0009**	0.19	0.6601	**5.80**	**0.0304**	0.07	0.7848	**18.27**	**<0.0001**	0.03	0.8562	**1.99**	**0.0235**
Y(II)	168	0.86	0.3559	2.29	0.1321	**7.65**	**0.0063**	0.15	0.7058	2.20	0.1402	1.22	0.2706	2.19	0.1409	**1.73**	**0.0421**
ETR(II)	168	0.86	0.3556	2.33	0.1291	**7.65**	**0.0063**	0.15	0.7026	2.19	0.1410	1.23	0.2685	2.21	0.1388	**1.73**	**0.042**
qN	168	**25.48**	**<0.0001**	1.83	0.1781	3.68	0.0569	3.08	0.1010	**4.23**	**0.0413**	**8.29**	**0.0045**	1.54	0.2156	**1.99**	**0.0234**
qP	168	2.98	0.0863	**8.05**	**0.0051**	**10.04**	**0.0018**	<0.01	0.9581	**4.41**	**0.0373**	2.48	0.1173	2.83	0.0941	1.53	0.063
**Antioxidant variables**																	
T_AOC (U g^−1^)	170	**40.69**	**<0.0001**	**18.35**	**<0.0001**	1.20	0.2742	0.66	0.4307	0.03	0.8559	0.01	0.9146	0.03	0.8592	**1.99**	**0.0231**
SOD (U g^−1^)	170	0.84	0.3603	0.33	0.5666	0.75	0.3883	0.78	0.3919	0.57	0.4531	<0.01	0.9705	0.14	0.7112	**2.08**	**0.0186**
H_2_O_2_ (µmol g^−1^)	170	**6.83**	**0.0098**	**18.39**	**<0.0001**	0.56	0.4559	6.14	**0.0266**	0.09	0.7595	0.13	0.7200	0.10	0.7485	**1.96**	**0.0252**
$O_{2}^{-}\cdot$ (nmol g^−1^)	170	**19.65**	**<0.0001**	**26.32**	**<0.0001**	3.47	0.0644	2.07	0.1723	<0.01	0.9603	0.05	0.8192	0.01	0.9343	**2.02**	**0.0219**
${\text{S}}_{\cdot OH}$ (%)	170	0.57	0.4531	**12.13**	**0.0006**	0.18	0.6709	2.55	0.1325	0.12	0.7278	0.010	0.9061	0.15	0.7007	1.37	0.1749

Significant results are shown in bold.

#### PSI parameters.

**Figure 5.**
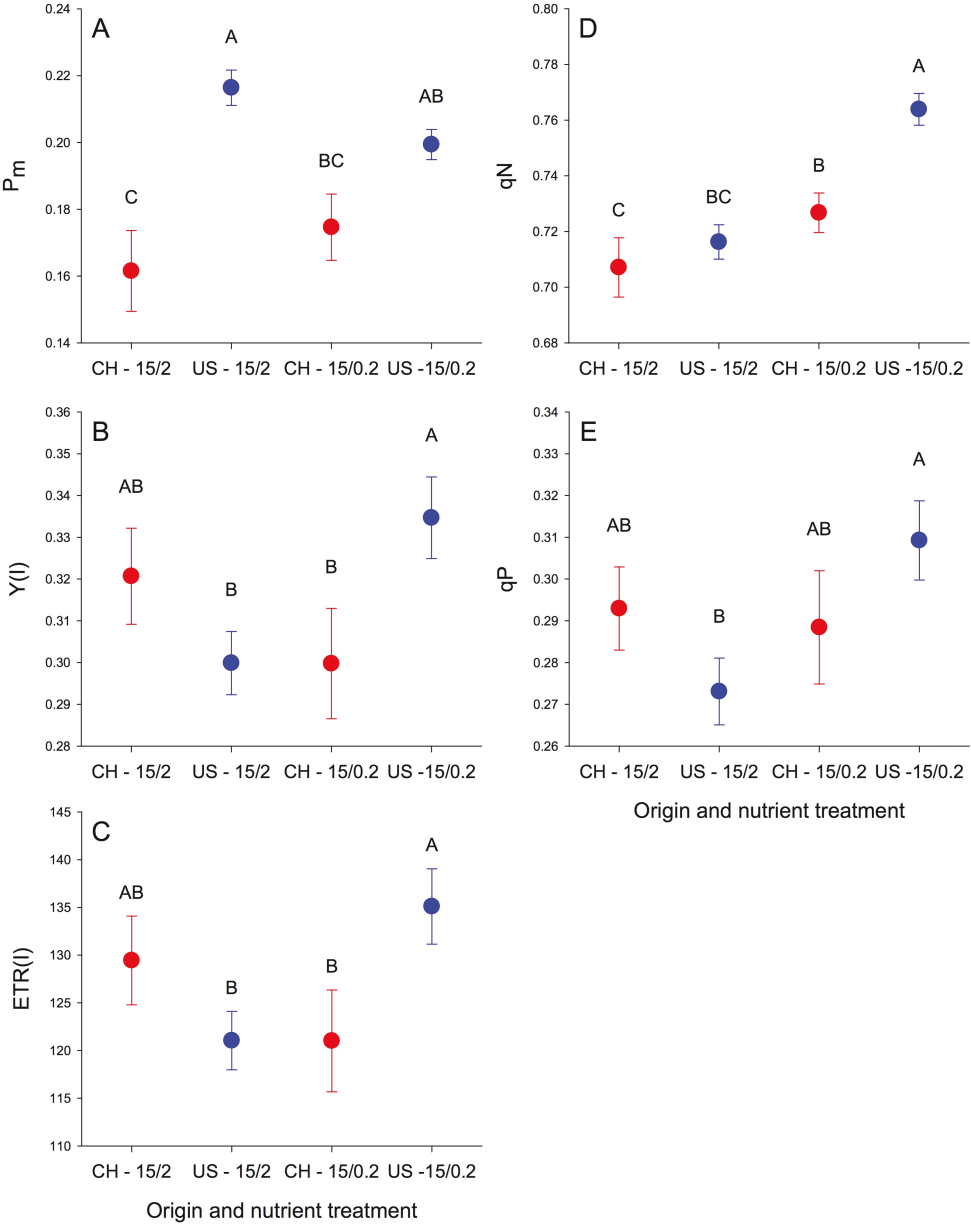
Effect of origin and nutrient treatment on Pm (A), Y(I) (B), ETR(I)(C), qN(D) and qP (E) in introduced and native populations in N:P and water treatment experiment (experiment 2).

*P*_m_ was higher for US populations at NP 15:2 but US populations tended to decrease and China populations tended to increase at NP 15:0.2 so they were comparable with lower P (Table 2, Fig. 1D, Fig. 5A). *P*_m_ also increased with salinity. Y(I) and ETR(I) tended to be higher for China populations at NP 15:2 but US populations were significantly higher at NP 15:0.2 (Table 2, Fig. 1E and F). Y(I) and ETR (I) also depended on NP × salinity with each being highest at NP 15:0.2 and water, intermediate at NP 15:2 and saline and lower in the other two combinations (Fig. 5B and C).

#### PSII parameters.

**Figure 6.**
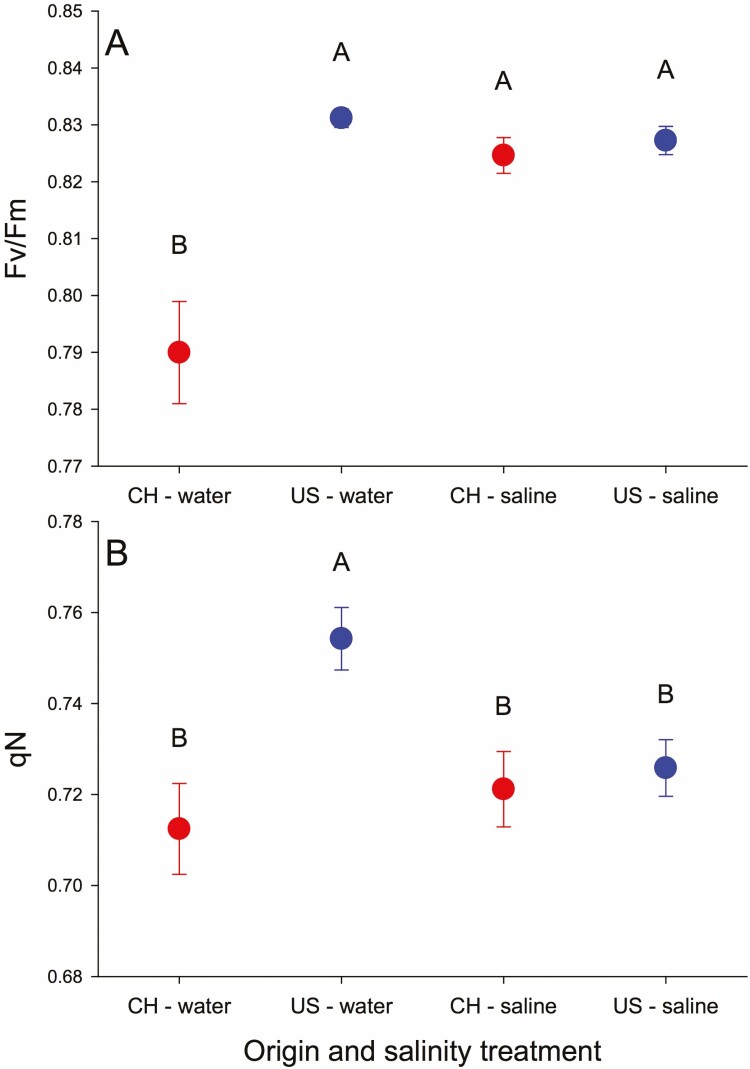
Effect of origin and salt stress on *F*_v_/*F*_m_ (A) and qN (B) in introduced and native populations in N:P and water treatment experiment(experiment 2).

*F*_v_/*F*_m_ was low for China populations in water compared to other combinations of origin and salinity (Table 2, Fig. 3A, Fig. 6A). Y(II) and ETR(II) depended on NP × salinity with each being highest at NP 15:0.2 and water, intermediate at NP 15:2 and saline and lower in the other two combinations (Fig. 3G and H). US and China populations both had lower qN at NP 15:2 but US populations increased more at NP 15:0.2 so they were higher than China populations (Table 2, Fig. 3I, Fig. 5D). US populations in water had higher qN than other origin × salinity combinations (Fig. 6B). US populations had high qP at NP 15:0.2 with salinity and low qP in NP 15:2 and water with China populations intermediate (Table 2, Fig. 3J, Fig. 5E). qP also depended on NP × salinity in a pattern similar to Y(II) and ETR(II).

#### AOX parameters.

T-AOC, H_2_O_2_, $O_{2}^{-}\cdot$ and ${\text{S}}_{\cdot OH}$ all were higher at NP 15:0.2 with saline (Table 2, Fig. 4F–J). SOD did not depend on any factors (Table 2, Fig. 4G).

#### Correlations among variables.

All the mass variables were strongly positively correlated with each other. Mass variables were most positively correlated with PSI variables Pm, PSII variables *F*_v_/*F*_m_ and the antioxidant variables ${\text{S}}_{\cdot OH}$. The PSI variables Y(I) and ETR(I) were strongly positively correlated. The PSII variables Y(II), ETR(II) and qP were strongly positively correlated. The antioxidant variables T-AOC and H_2_O_2_ were strongly positively correlated.

## Discussion

Previous studies have reported the detrimental effects of nutrient deficiency and salinity on invasive plants, particularly in terms of their growth and photosynthetic phenotype characteristics (Funk and Vitousek 2007; Funk 2013; Pintó-Marijuan and Munné-Bosch 2013; Yang *et al.* 2015a, b, 2022; Guo *et al.* 2023). They also indicated that an appropriate amount of salt has a promoting effect on the growth and development of plants, enhancing their stress resistance (Li *et al.* 2015; Liu *et al.* 2023). This research in seedlings emphasizes how the better-photosynthetic-tolerant introduced populations outperform the native populations using chlorophyll fluorescence and antioxidant performance under different nutrient availability or combined with mild salinity conditions. We found that the chlorophyll fluorescence performance of *T. sebifera* depended on origin, nutrient levels and salinity. However, although the introduced populations showed obvious growth advantages with higher *F*_v_/*F*_m_ and *P*_m_, it did not demonstrate significant antioxidant advantages except demonstrating less accumulation of H_2_O_2_, when faced with nutrient deficiency/high N:P or mild salinity compared with native populations. Therefore, this research provides a valuable dataset for investigating adaptive strategies to nutrient deficiency/NP ratio or salinity habits associated with plant invasions.

### Introduced populations exhibit stronger nutrient deficiency inhibition without salinity, but with less H_2_O_2_ accumulating

Biomass accumulation and leaf area enlargement were inhibited significantly in both introduced and native populations by nutrient deficiency. However, biomass and leaf area in introduced populations were obviously greater than those in native populations regardless of treatments. These results are consistent with the findings of Funk (2013) and supported by other studies on *T. sebifera* (Yang *et al.* 2015a; Deng *et al.* 2019). A lack of sufficient macro- and micronutrients may decrease chlorophyll contents and inhibit the number of photosynthetic apparatus (Qi *et al.* 2013; Li and Sun 2016), which, in turn, reduces the photochemical efficiency and quantum yield of PSII and PSI by changing electron transfer pathway (such as from cyclic to pseudocyclic electron transport) or hindering electron transfer to the electron transport chain (Misra *et al.* 2012; Li and Sun 2016; Latifinia and Eisvand 2022), as shown in Fig. 1 and Fig. 3, subsequently leading to a decrease in biomass. Notably, there was a considerable decrease in *F*_v_/*F*_m_ and *P*_m_ caused by nutrient deficiency in introduced populations but not in native populations, indicating that introduced populations experienced greater inhibition (Fig. 2). On the other hand, the changing of electron transport may result in excessive production of $O_{2}^{-}\cdot$, which is converted to H_2_O_2_, ultimately leading to H_2_O_2_ accumulation (Zahra *et al.* 2022). Nevertheless, there was less H_2_O_2_ accumulation in introduced populations. It is probably because they were able to compensate for the negative effects by generating a greater thermal dissipation (qN, Fig. 3D) and faster or more efficient removal of H_2_O_2_, such as higher ${\text{S}}_{\cdot OH}$ in an introduced population (Fig. 4E), which strengthened self-protection and reduced damage to the photosynthetic membrane, and allowed for larger relative growth (Takahashi and Badger 2011; Pintó-Marijuan and Munné-Bosch 2013). Simultaneously, a similar increase in T-AOC, SOD and $O_{2}^{-}\cdot$ between introduced and native populations suggests that introduced and native *T. sebifera* plants employ similar SOD strategies but different H_2_O_2_ scavenge pathways when faced with nutrient deficiency.

### Introduced populations were more adapted to NP15:2, while native populations were more adapted to NP15:0.2 without salinity

When there was no salinity, compared with NP15:2, NP15:0.2 promoted a significant increase in the biomass and leaf area of native populations, but brought about a significant decrease in leaf area for introduced populations, although NP15:0.2 increased the photosynthetic quantum yield and electron transfer rate for both populations. This suggests that introduced populations were better adapted to NP15:2, while native populations were better adapted to NP15:0.2. The slight increase in T-AOC, H_2_O_2_ and $O_{2}^{-}\cdot$ under NP15:0.2 and increased qN reflected a self-protective mechanism against phosphorus deficiency in plants for both populations (Goussi *et al.* 2021). Unutilized light energy is dissipated through heat, and electrons are unable to fully transfer to the corresponding acceptor, such as NADPH, leading to pseudocyclic electron transport or other electron transport types, and subsequent accumulation of ROS (H_2_O_2_ and $O_{2}^{-}\cdot$) under NP15:0.2 (Asada 2006; Sharma *et al.* 2012; Bose *et al.* 2014; Rajput *et al.* 2021), especially for native population because of their slight lower ${\text{S}}_{\cdot OH}$ (Fig. 4J). The increased photosynthetic quantum yield and electron transfer rate under NP15:0.2 may be due to an increase in chlororespiration (Wiciarz *et al.* 2015) stimulated by less phosphorus. Simultaneously, NP15:2 may provide additional phosphorus to support RNA synthesis in introduced populations. This preference for phosphorus can aid in the maintenance of introduced plant mass and larger leaf area (Rivas-Ubach *et al.* 2012; Esterhuizen *et al.* 2020).

Introduced populations subjected to growth inhibition induced by mild salinity show lower H_2_O_2_ accumulation and better growth than native populations.

Our results are in agreement with those of previous studies showing inhibition of *T. sebifera* mass accumulation and leaf area expansion under salt stress regardless of nutrient/N:P level (Chen *et al.* 2013; Yang *et al.* 2015a, b, 2022). This could be attributed to a decrease in cell growth expansion caused by a reduction in turgor pressure resulting from decreased water availability to the roots (Goussi *et al.* 2021). These studies also demonstrated a high tolerance of introduced populations of Chinese tallow tree to mild salinity (Chen *et al.* 2013; Yang *et al.* 2015a, b, 2022), since salinity can enhance the net positive effects of this species on soil, particularly in introduced populations. In the two experiments here, the lower H_2_O_2_ accumulation and slightly higher ${\text{S}}_{\cdot OH}$ of introduced populations, suggesting better ROS scavenging and better growth, especially in high nutrient conditions, further support this conclusion. The results are similar to those of an interspecific study by Musso *et al.* (2021) in which an invasive species (*M. minutiflora*) showed a higher effect of water than a native species (*S. microstachyum*) when soil nutrients were more abundant. However, chlorophyll fluorescence performance of *T. sebifera* under salinity depended on origin and nutrient/N:P, as discussed below.

### Mild salt induces more limited chlorophyll fluorescence changes in introduced populations than in native ones under high nutrient levels

Under high nutrient conditions, mild salinity stimulated photosynthetic electron transport and photosynthetic quantum yield for both introduced and native populations, possibly due to pronounced ROS production (particularly H_2_O_2_) from the plastoquinone pool and increased chlororespiration (Wiciarz *et al.* 2015; Manaa *et al.* 2019; Goussi *et al.* 2021) or slight incline of the rates of energy capture and dissipated energy flux (Goussi *et al.* 2021). Simultaneously, mild salt did not alter *F*_v_/*F*_m_ of introduced populations but it decreased *F*_v_/*F*_m_ of native populations. This may be due to salinity causing greater accumulation of H_2_O_2_ (Fig.4H), weaker ${\text{S}}_{\cdot OH}$ (Fig. 4J) and inhibiting PSII repair in native populations compared with introduced populations (Bose *et al.* 2014). Together with unchanged heat dissipation (qN, Fig. 3I) in introduced populations, these changes may reflect a physiological response of introduced plants to adapt to saline environments and maintain the stability of PSII and PSI (Goussi *et al.* 2021). By contrast, this suggests salt-induced more severe impact on plants of native population in nutrient-rich environments than on plants from introduced populations.

### Mild salt does not exacerbate the growth inhibition induced by low nutrients in both introduced and native populations

The responses of plants to combinations of stress factors are highly complex (Goussi *et al.* 2021). Compared with low-nutritve treatments, our experiment showed that salinity did not further enhance the growth inhibition caused by low nutrients. The increased *P*_m_, while other chlorophyll fluorescence parameters were unchanged in both introduced and native populations, suggests that electron transport to PSI was strengthened without affecting the photochemical ability of PSII (Huang *et al.* 2015). This may be caused by slight damage to the photosynthetic process due to an imbalance in Na^+^ ions (Acosta-Motos *et al.* 2017). It may also indicate why the plants still showed a slight increase in T-AOC and accumulation of $O_{2}^{-}\cdot$ and H_2_O_2_, especially in the native population. Other scavengers may function in mesophyll cells (Morais *et al.* 2012). Previous research on native vs. invasive species confirmed that under salt stress, invasive *Acacia longifolia* was more effective than native *Ulex europaeus* in improving adaptability to adverse environments (Morais *et al.* 2012). However, in our intraspecific study, introduced populations did not exhibit better performance under salt stress and nutrient deficiency except for lower H_2_O_2_ accumulation. Therefore, we speculate that a similar antioxidant strategy in both populations may explain why mild salinity did not further exacerbate the low nutrient-induced inhibition.

### Mild salt-induced a negative effect only in native populations under NP15:0.2

Under NP15:2, salt treatment only increased *P*_m_ and caused fluctuations in *F*_v_/*F*_m_ in native populations while all other chlorophyll fluorescence parameters were unaffected. The increase in *P*_m_ might be due to the enlargement of the active reaction centre antennae induced by salinity, as discussed above (salt treatment under high nutrients; Goussi *et al.* 2021). In addition, salt ions such as Na^+^ have different effects on the degree of opening of the PSII reaction centre, and can decrease *F*_0_ (from 0.203 ± 0.004 to 0.188 ± 0.003) in native populations.

On the other hand, Oliveira *et al.* (2014) reported that native *Anadenanthera colubrina* and invasive *Prosopis juliflflora* showed different responses to leaf phosphorus supply under water stress for several measured variables. In our experiment, only under NP15:0.2 were similar conclusions obtained for both native and introduced populations of Chinese tallow trees. Under NP15:0.2, the decrease in photosynthetic electron transport rate and photosynthetic quantum yield only in native population plants induced by salinity suggests that salinity has a stronger negative effect on native populations than introduced populations. Slightly less H_2_O_2_ and $O_{2}^{-}\cdot$ contents and higher ${\text{S}}_{\cdot OH}$ in introduced populations, meaning less damage by salinity, further supports this conclusion (Mittler *et al.* 2022). Together, these findings indicate that introduced populations were adapted better to salinity under NP15:0.2, which might also be related to stronger symbiosis with arbuscular mycorrhizal fungi (AMF), as demonstrated for introduced *T. sebifera* in which salt promoted symbiosis with AMF and improved resistance (Yang *et al.* 2015a, b).

## Conclusion

Our results indicate that nutrient deficiency caused more severe growth inhibition in both introduced and native *T. sebifera* populations compared with mild salinity, though both nutrient deficiency and salinity lowered ETR and photochemical quantum yield (Yield), while high N:P (15.0.2) had no such effect. Introduced population plants were larger, partly due to their higher *F*_v_/*F*_m_ and *P*_m_, especially under high nutrient and either N:P conditions. Increased ETR and Yield of introduced population plants under combined treatment of salinity with high nutrient or high N:P (15.0.2) might be responsible for its better growth. However, introduced populations appear to have a better capacity for resisting salinity rather than nutrient shortage. Furthermore, total antioxidant capacity was similar between introduced and native populations apart from lower H_2_O_2_ in introduced population plants, which seems to be a key factor for the better adaptation. Though qN was higher in introduced populations implying stronger heat dissipation capacity, it did not have a significant relationship with biomass. These findings suggest that chlorophyll fluorescence and H_2_O_2_ advantages in introduced seedlings might contribute to their better growth when invading nutrient-rich or low N:P conditions or in combination with mild salinity environments. So the stronger photosynthetic capacity in the seedlings of the introduced *T. sebifera*, coupled with low levels of oxidative damage, can provide a solid physiological foundation for being tree.

## Supporting Information

The following additional information is available in the online version of this article –

**Figure S1.** Mass on introduced and native populations under nutrient deficit and water treatment experiment (experiment 1, A-D) and N:P and water treatment experiment (experiment 2, E-H). Asterisk represents significant differences between US and CH populations at the same treatment level. Fig E-H unmarked the letters because the interactions between treatments were not significant.

**Figure S2.** Correlations between parameters in nutrient deficit and water treatment experiment (experiment 1) (A) and N:P and water treatment experiment (experiment 2) (B).

**Table S1.** Populations used in two experiments.

**Data files** supporting the findings of this study

plae024_suppl_Supplementary_Table_S1_Figures_S1-S2

plae024_suppl_Supplementary_Data

## Data Availability

All data supporting the findings of this study are available as supporting information.
