# Supplementary material for: Chlorophyll fluorescence characteristics and H2O2 contents of Chinese tallow tree are dependent on population origin, nutrients and salinity
Source: AoB Plants. 2024 May 2;16(3):plae024. doi: 10.1093/aobpla/plae024 (PMC11285151; doi:10.1093/aobpla/plae024)
Supplement: plae024_suppl_Supplementary_Table_S1_Figures_S1-S2 [file plae024_suppl_supplementary_table_s1_figures_s1-s2.zip › Supporting information.pdf]

Supplementary material

Table S1 Populations used in two experiments

Code	Collection location	Latitude	Longitude
China populations			
DW	Dawu (Hubei)	31°35′ N	114°14′ E
GL	Guilin (Guangxi)	25°04′ N	110°18′ E
HC	Hongcun (Anhui)	30°00′ N	117°59′ E
HF	Hefei (Anhui)	31°50′ N	117°09′ E
HS	Miluo (Hunan)	28°53′ N	113°12′ E
WX	Wuxi (Jiangsu)	31°36′ N	120°14′ E
YS	Yingtan (Jiangxi)	28°19′ N	117°03′ E
ZS	Zhangshu (Jiangxi)	28°02′ N	115°25′ E
US populations			
GA1	Hutchinson Island (Georgia)	32°06′ N	81°06′ W
GA3	Lake Charles (Louisiana)	30°14′ N	93°09′ W
LA1	Pumpkin Center, Louisiana	30°28′ N	90°32′ W
LA6	Limehouse, South Carolina	32°09′ N	81°06′ W
MS1	Moss Point, Mississippi	30°26′ N	88°31′ W
TX1	Houston (Texas)	29°47′ N	95°02′ W
TX2	La Marque (Texas)	29°22′ N	95°02′ W
TX5	Port Arthur (Texas)	29°53′ N	94°02′ W

Figure S1

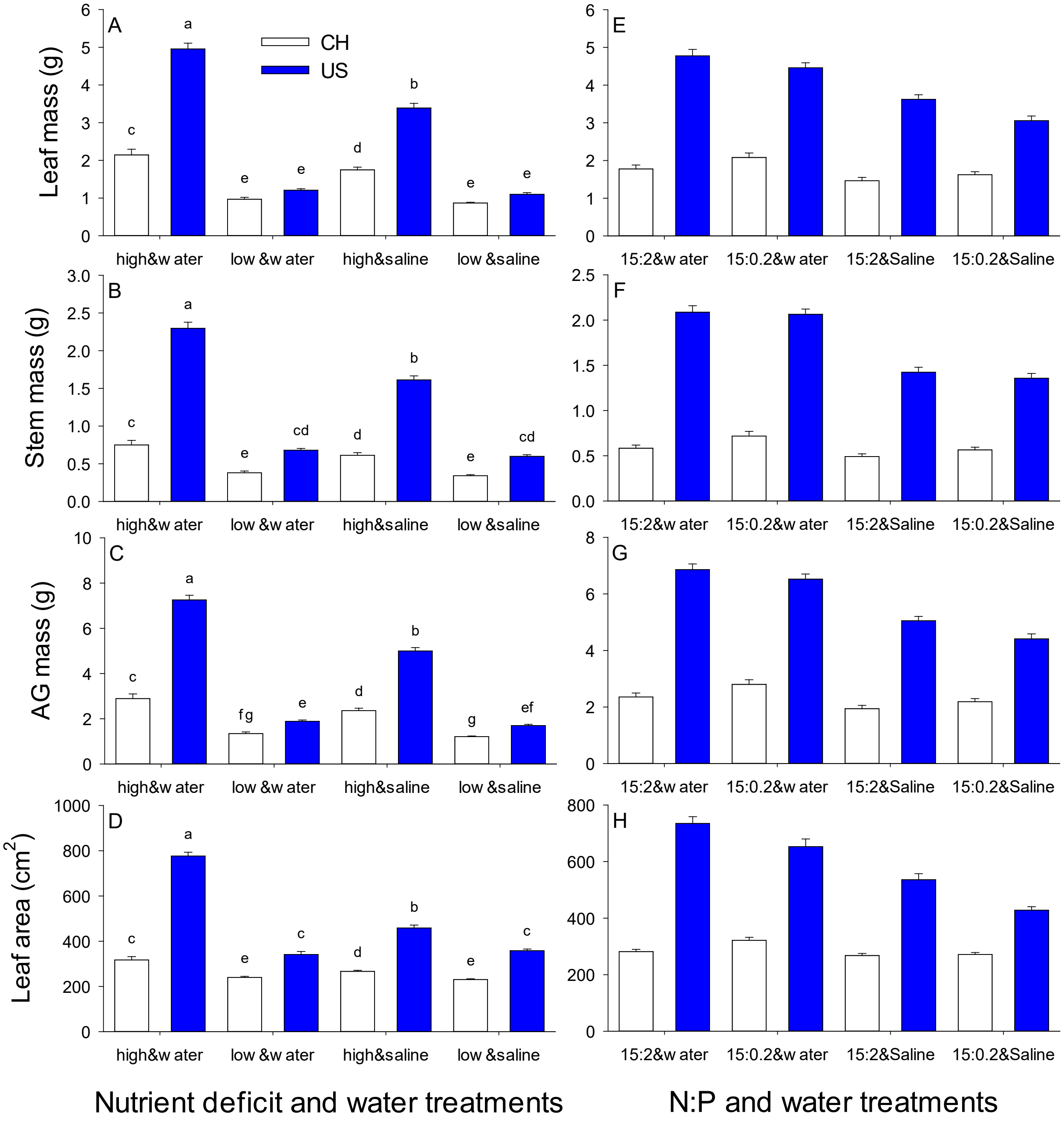

Figure S1 Mass on introduced (blue) and native (white) populations under nutrient deficit and water treatment experiment (experiment 1, A-D) and N:P and water treatment experiment (experiment 2, E-H).

Figure S2

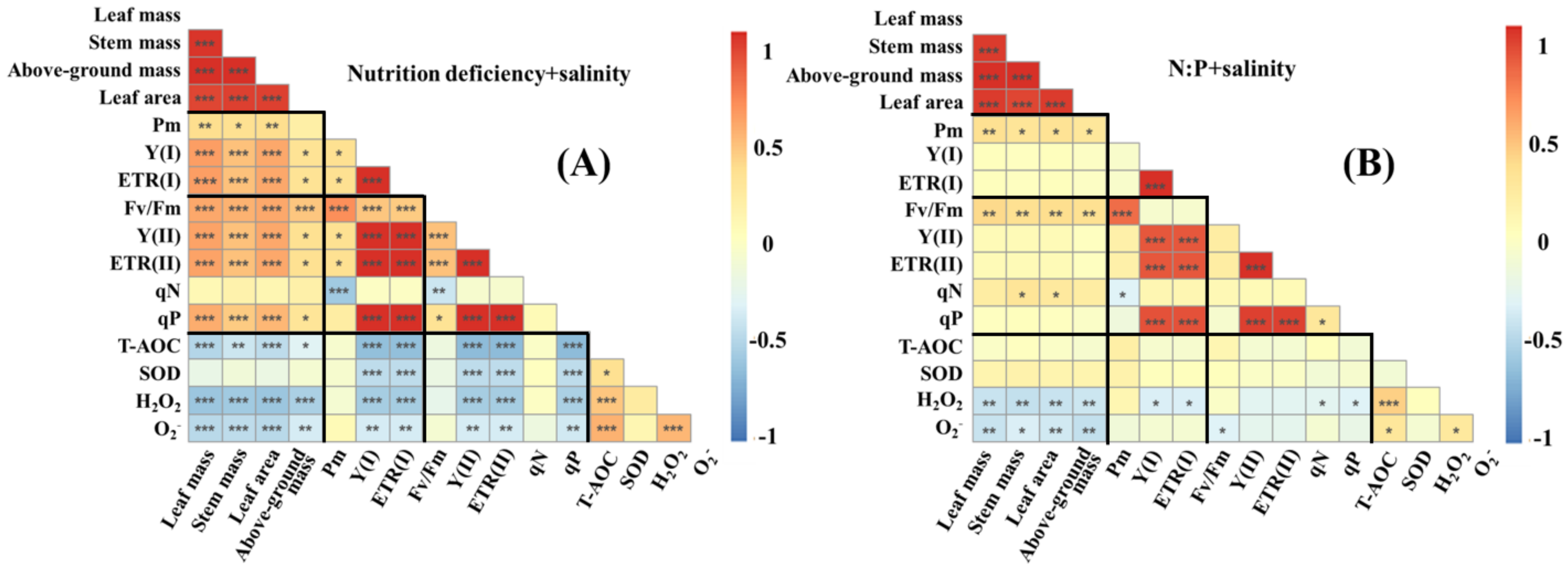

Figure S2 Correlations between parameters in nutrient deficit and water treatment experiment (experiment 1) (A) and N:P and water treatment experiment (experiment 2) (B)
