# Supplementary material for: Chlorophyll fluorescence characteristics and H2O2 contents of Chinese tallow tree are dependent on population origin, nutrients and salinity
Source: AoB Plants. 2024 May 2;16(3):plae024. doi: 10.1093/aobpla/plae024 (PMC11285151; doi:10.1093/aobpla/plae024)
Supplement: plae024_suppl_Supplementary_Table_S1_Figures_S1-S2 [file plae024_suppl_supplementary_table_s1_figures_s1-s2.zip › Table SI.docx]

**Table S1** Populations used in two experiments

| **Code** | **Collection location** | **Latitude** | **Longitude** |
| --- | --- | --- | --- |
| China populations | | | |
| DW | Dawu (Hubei) | 31°35′ N | 114°14′ E |
| GL | Guilin (Guangxi) | 25°04′ N | 110°18′ E |
| HC | Hongcun (Anhui) | 30°00′ N | 117°59′ E |
| HF | Hefei (Anhui) | 31°50′ N | 117°09′ E |
| HS | Miluo (Hunan) | 28°53′ N | 113°12′ E |
| WX | Wuxi (Jiangsu) | 31°36′ N | 120°14′ E |
| YS | Yingtan (Jiangxi) | 28°19′ N | 117°03′ E |
| ZS | Zhangshu (Jiangxi) | 28°02′ N | 115°25′ E |
| US populations | | | |
| GA1 | Hutchinson Island (Georgia) | 32°06′ N | 81°06′ W |
| GA3 | Lake Charles (Louisiana) | 30°14′ N | 93°09′ W |
| LA1 | Pumpkin Center, Louisiana | 30°28′ N | 90°32′ W |
| LA6 | Limehouse, South Carolina | 32°09′ N | 81°06′ W |
| MS1 | Moss Point, Mississippi | 30°26′ N | 88°31′ W |
| TX1 | Houston (Texas) | 29°47′ N | 95°02′ W |
| TX2 | La Marque (Texas) | 29°22′ N | 95°02′ W |
| TX5 | Port Arthur (Texas) | 29°53′ N | 94°02′ W |
